# Novel Immune Mechanisms in Hypertension and Cardiovascular Risk

**DOI:** 10.1007/s12170-017-0537-6

**Published:** 2017-03-06

**Authors:** Ryszard Nosalski, Eilidh McGinnigle, Mateusz Siedlinski, Tomasz J. Guzik

**Affiliations:** 1 BHF Centre for Excellence Institute of Cardiovascular and Medical Sciences, University of Glasgow, Glasgow, Scotland UK; 2 Department of Internal and Agricultural Medicine, Faculty of Medicine, Jagiellonian University Medical College, Krakow, Poland

**Keywords:** Hypertension, Immune system, Inflammation, Cytokines, T cells, Vascular function, microRNA

## Abstract

**Purpose of Review:**

Hypertension is a common disorder with substantial impact on public health due to highly elevated cardiovascular risk. The mechanisms still remain unclear and treatments are not sufficient to reduce risk in majority of patients. Inflammatory mechanisms may provide an important mechanism linking hypertension and cardiovascular risk. We aim to review newly identified immune and inflammatory mechanisms of hypertension with focus on their potential therapeutic impact.

**Recent Findings:**

In addition to the established role of the vasculature, kidneys and central nervous system in pathogenesis of hypertension, low-grade inflammation contributes to this disorder as indicated by experimental models and GWAS studies pointing to SH2B3 immune gene as top key driver of hypertension. Immune responses in hypertension are greatly driven by neoantigens generated by oxidative stress and modulated by chemokines such as RANTES, IP-10 and microRNAs including miR-21 and miR-155 with other molecules under investigation. Cells of both innate and adoptive immune system infiltrate vasculature and kidneys, affecting their function by releasing pro-inflammatory mediators and reactive oxygen species.

**Summary:**

Immune and inflammatory mechanisms of hypertension provide a link between high blood pressure and increased cardiovascular risk, and reduction of blood pressure without attention to these underlying mechanisms is not sufficient to reduce risk.

## Introduction

Hypertension (HT) is a common disorder with substantial impact on public health because of its potential sequelae of stroke, heart failure and kidney disease; subsequently, it is a major source of morbidity and mortality [1]. It affects 30% of adults in Europe and USA with additional 30% at high risk of the disease [2], and importantly, its prevalence increases with age [3].

Despite the progress in its diagnosis and treatment, the aetiology of HT remains unclear and a matter of substantial debate. It is widely acknowledged that function of the vascular system, kidneys and sympathetic nervous system is critical for control and maintenance of blood pressure (BP) [1]. Vascular resistance, stiffness and remodelling as well endothelial dysfunction are hallmarks of HT [4–7]. Kidney transplantation from hypertensive donors rises BP in recipients in animal and human studies [8, 9]. Sympathetic nervous system hyperactivity contributes to initiation, maintenance and progression of HT [10]. Furthermore, deletion of extracellular superoxide dismutase or administration of angiotensin II (Ang II) into circumventricular organs (CVO) raises blood pressure, while lesions of these regions prevent experimental HT [11, 12].

## Inflammation—Hallmark of Hypertension

In addition to established roles of the vasculature, kidneys and central nervous system, there is a mounting evidence, to suggest that chronic low-grade inflammation contributes to cardiovascular disease (CVD) [13••, 14], including HT [15]. For instance, C-reactive protein (CRP) is a prototypic marker of inflammation which is elevated in many CVD such as acute myocardial infraction, coronary artery disease and HT [13••, 16]. Vongpatanasin et al. have shown that modest elevations in CRP are sufficient not only to increase BP but also exaggerate response to Ang II [17]. Furthermore, human and animal studies emphasize impact of CRP on endothelial vasodilator function [18, 19] via direct action on endothelial nitric oxide synthase (eNOS) and/or activation of vascular NADPH oxidases leading to reactive oxygen species (ROS) formation [19–21]. In addition to CRP, the inflammatory process involves a complex interplay between cells and pro-inflammatory cytokines. This process is strictly controlled by the immune system. Various cells of the innate and adaptive immune systems contribute to initiation and maintenance of inflammation. Furthermore, resolution of inflammation occurs by different mechanism in many CVD, including HT.

## Link Between Immune Cells and Hypertension

The concept that the immune system contributes to hypertension is not new. Fifty years ago, the pioneering study of White and Grollman described the role of immunosuppressive therapy on BP levels in rats with kidney infarction [22]. Further studies have shown that the transfer of immune cells isolated from lymph nodes or spleen of hypertensive animals increases BP in normotensive recipients [23, 24]. Moreover, thymectomy of deoxycorticosterone acate (DOCA)-salt-treated mice [25] or spontaneously hypertensive rats [26] attenuates experimental HT. Despite the above-mentioned studies showing the potential role of the immune system in HT, lack of advanced animal models and methodology did not allow for a more thorough understanding this phenomenon.

### T Cells

In 2007, the landmark study by Guzik et al. demonstrated that HT induced by Ang II or DOCA salt is blunted in RAG1^−/−^ mice lacking functional lymphocytes, and the hypertensive response is restored by adoptive transfer of T but not B lymphocytes [27]. These results were subsequently confirmed by Crowley et al., who showed that T cells are essential for the full development of AngII-dependent HT in immunodeficient *scid* mice [28] and by Mattson et al., who demonstrated a similar phenomenon in RAG1^−/−^ rats [29]. In addition to this, mycophenolate immunosuppressive therapy which, inhibits T cell proliferation, reduces BP in Dahl salt-sensitive rats [30]. It is known that Ang II acts through the AT1 and AT2 receptors, both of which are present on the surface of T cells [26]. Furthermore, adoptive transfer of AT1 receptor deficient T cells into RAG1^−/−^ animals leads to a blunted hypertensive response in Ang II-induced hypertension [27]. Infusion of Ang II increases the percentage of circulating T cells with effector phenotype (CD69+, CD25+, CCR5+) in both *in vivo* and *in vitro* studies [27, 31]. Additionally, T cells with effector phenotype accumulate in perivascular adipose tissue (PVAT) and kidneys, and affect endothelial function and vascular fibrosis [30–32, 33•]. Interestingly, a meta-analysis of GWAS data pointed polymorphisms in the *SH2B3* gene as significant predictors of systolic and diastolic BP. This gene encodes for the lymphocyte adaptor protein, *lnk* [34]. Keeping with this, Saleh et al. have shown that the loss of *lnk* exacerbates Ang II-induced HT and its associated renal and vascular dysfunction. Moreover, *lnk*-deficient mice have increased T cell activation and cytokine production in comparison to wild-type animals [35]. Higher cytokine production by immunosenescent cytotoxic CD8+ T cells (CD28 null and positive for CD57) as well as augmentation of their number have been previously reported in hypertensive patients [36].

Among T cells, there is a distinct subset of regulatory cells. They express CD4 and CD25 surface markers and a forkhead transcription factor 3 (FOXP3). This unique subset possesses the capacity to suppress innate and adaptive immune responses [37]. Experimental HT is related to a decline in the number of Treg cells in *in vitro* and *in vivo* studies [38, 39]. Recent studies have shown that adoptive transfer of Treg cells reduces blood pressure [40, 41] and ameliorates endothelial function in Ang II-treated animals [39]. Further studies have shown this is accompanied by the attenuation of NADPH oxidase activity, which is critical in the development of vascular dysfunction [42, 43].

### B Cells

Clinical and experimental HT is associated with raised serum IgG, IgA or IgM antibodies produced by B cells [44]. Although, transfer of B cells did not restore HT in Ang II-infused RAG1^−/−^ mice [27], B cell activation does appear to be dependent on highly specific interactions with T cells [45] which are absent in RAG1^−/−^ animals. Recently, Drummond’s group has shown that Ang II infusion leads to increased production of antibodies by activated B cells. Genetic deficiency of B-cell-activating factor receptor, or pharmacological depletion of B cells, protects against BP elevation and the end organ sequelae of Ang II such as collagen deposition and aortic stiffness. These effects are restored by the adoptive transfer of B cells [46].

### Monocytes and Macrophages

Monocytes and macrophages have been implicated in various models of experimental HT [47, 48]. Ang II-induced HT is associated with an increased number of circulating monocytes [49, 50], and their elimination leads to decreased severity of HT, associated reduction of vascular ROS generation and improvement of vascular function [49]. Monocytes are circulating precursors of macrophages, which accumulate in the PVAT, adventitia and kidneys during HT [4, 50, 51]. Infiltrating macrophages release pro-inflammatory mediators and produce free radicals via NOX2 NADPH oxidase that changes vascular homeostasis [52–54]. Macrophage colony-stimulating factor (m-CSF) deficiency is associated with attenuated Ang II-induced HT, arterial remodelling, endothelial dysfunction, superoxide generation, NADPH oxidase activation and vascular inflammation [47]. Correspondingly, pharmacological blockade of macrophage CCR2 receptors, using INCB3344, prevents macrophage accumulation and reverses DOCA salt and Ang II-induced HT [50, 51].

Toll-like receptors (TLRs) have an important role in the activation of macrophages and monocytes [55]. They provoke cytokine and chemokine production through activation of NF-κB (nuclear factor kappa B) [56]. TLR4 is upregulated in Ang II-induced HT. Anti-TLR4 antibody treatment normalises BP and reduces inflammation and vascular changes associated with HT through MyD88-dependent activation and JNK/NF-κB signalling pathway [57]. Similarly, neutralization of TLR4 reduces BP and augmented vascular contractility in adult spontaneously hypertensive rats [58]. Finally, upon activation, macrophages and monocytes can activate T cells via antigen presentation, expression of costimulatory ligands and release of mediators that modulate their function and/or chemotaxis [53, 55].

### Dendritic Cells

Evidence suggests that dendritic cells (DCs) play a role in the development of HT. DCs from hypertensive animals produce an increased amount of superoxide and a wide range of cytokines (IL-1β, IL-6, IL-23), which affect T cell polarization into the inflammatory phenotype [59]. Transfer of DCs from hypertensive donor mice into C57BL/6 mice results in the development of severe HT in response to sub-pressor dose of Ang II while having no effect in mice that received DCs from control animals [59]. Hypertensive mice have demonstrated higher levels of DCs with increased expression of costimulatory ligands CD80 and CD86, which are the hallmark of their activation [60]. Moreover, treatment with the pharmacological agent CTLA4-Ig (blocking B7-dependent costimulation) resulted in reduction of BP in both Ang II and DOCA salt-induced HT [60]. Chronic oxidative stress, associated with HT, leads to formation of immunogenic isoketal-protein adducts, which accumulate in DCs and promote T cell activation. Interestingly, increased isoketal adducts are also observed in immune cells of hypertensive patients [59].

### Natural Killer Cells

Studies of Taherzadeh et al. have shown that NK gene complex is an important determinant to genetically determined sensitivity to develop HT and associated vascular remodelling in L-NAME-induced HT in mice [61]. An increased number of NK cells are also observed in the circulation of pregnant hypertensive rats [62]. Moreover, depletion of NK cells leads to protection from Ang II-induced vascular dysfunction [63].

### Neutrophils

There are inconsistent results from studies investigating the role of neutrophils in HT. Pharmacological depletion of this subpopulation of leukocytes was associated with a significant fall in systolic BP in vivo and an attenuation in phenylephrine-induced vasoconstriction [64]. Conversely, selective depletion of circulating neutrophils protected against oxidative stress but not against the development of Ang II-induced HT [65]. Similarly, restoration of neutrophils in LysM^iDTR^-depleted mice with monocytes did not restore pathophysiological action of Ang II [49].

## Cytokines as Key Mediators in Hypertension

**Fig. 1:**
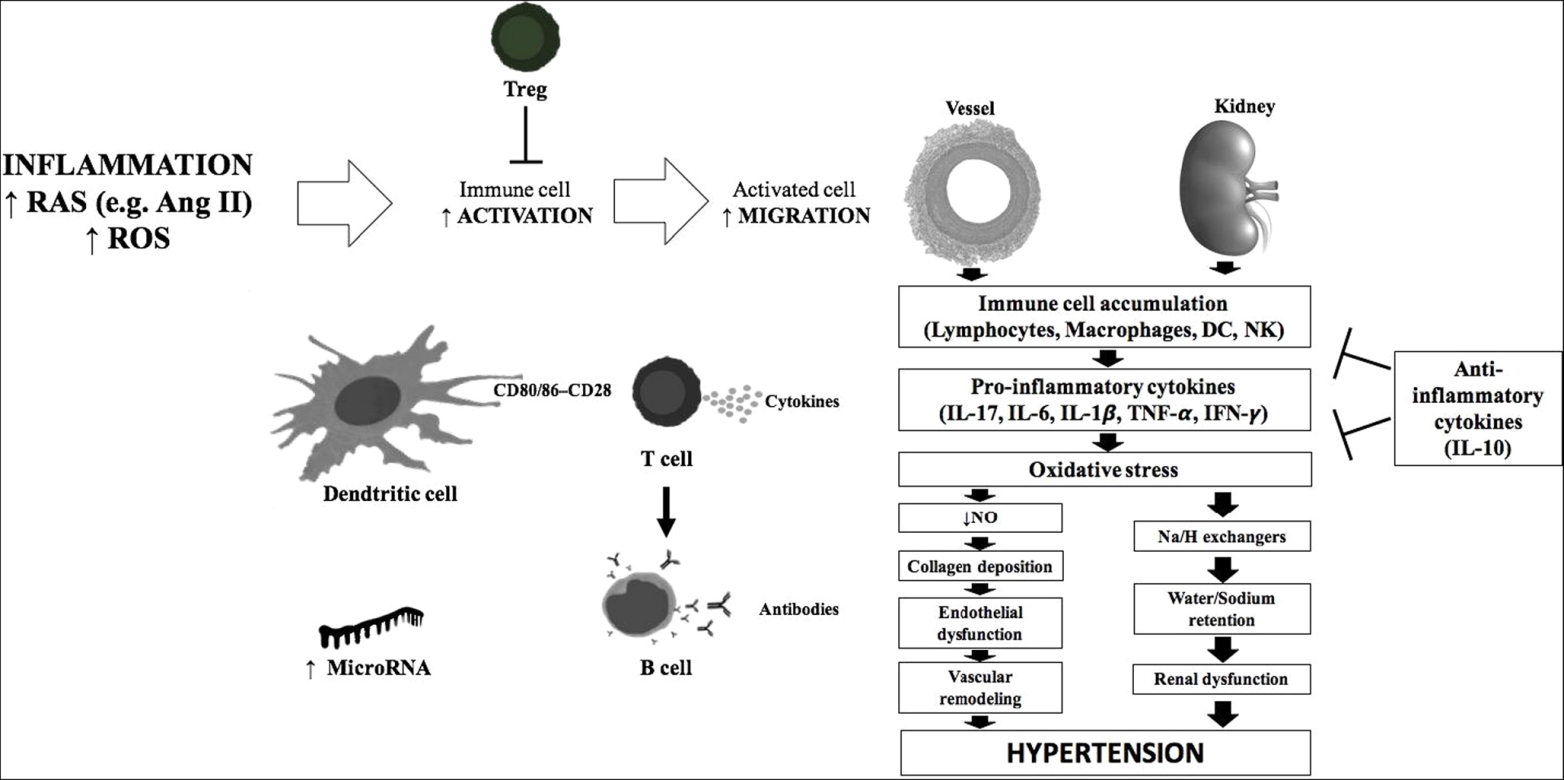
Immune mechanism of hypertension

During progression of HT, immune cells accumulate in target organs, of which kidneys and the vasculature are particularly vulnerable [4, 33•, 66] (Figure 1). These cells produce potent cytokines that affect vascular and renal function, which are essential for the development of HT. In recent years, numerous cytokines with a crucial role in HT have been reported.

### Tumour Necrosis Factor Alpha

Tumour necrosis factor alpha (TNF-α) is produced by many cell types including immune cells, vascular cells and adipocytes [67]. Various studies have shown that HT is associated with elevated production of TNF-α by different immune cells and a subsequent rise is observed in the circulation [27, 62, 68, 69]. Blockade of AT1 receptors in patients with HT results in a significant reduction of circulating levels of TNF-α. Mice lacking TNF-α gene or mice treated with etanercept (TNF-α antagonist) do not develop HT in response to Ang II [27, 68]. Replacement therapy with recombinant TNF-α restores action of Ang II [68]. Stimulation of endothelial cells with TNF-α decrease eNOS expression [70] by destabilisation of eNOS mRNA [71], which impairs ability of ECs to produce NO. TNF-α activates NF-κB and NADPH oxidase [70], which play an important role in the induction of oxidative stress and overexpression of both chemokines and adhesion molecules [68].

### Interferon Gamma

Another pro-inflammatory cytokine produced by various immune cells which plays a role in HT is interferon gamma (IFN-γ) [63, 72]. Experimental HT is associated with increased production of IFN-γ by activated T cells and NK cells [4, 35, 63]. The *knock down* of IFN-γ results in a blunted increase of Ang II-induced murine BP [35]. In contrast, this phenomenon is not observed in IFN-γ receptor-1-deficient mice [73]. Loss of *lnk* exacerbates production of IFN-γ by CD8+ lymphocytes as well as enhances impairment of endothelial-dependent relaxation as compared to wild-type mice [35]. Interestingly, incubation of aortic segments with IFN-γ *ex vivo* promotes endothelial dysfunction that is partially reversed by preincubation with PEG-SOD [4], which ameliorates oxidative stress in vasculature [74]. IFN-γ also has a strong impact on superoxide production via upregulation of the expression and activity of NADPH oxidases in human aortic smooth muscle cells [75]. It acts directly on VSMC to induce their proliferation and apoptosis [76, 77]. Furthermore, neutralization of IFN-γ biologic action prevents outward vascular remodelling of human coronary arteries induced by allogenic T cells in SCID/beige mice [78]. IFN-γ affects the RAS system as well as sodium-proton-exchanger type 3 transporter in the kidneys, which leads to increased production of angiotensinogen and modulation of sodium absorption, respectively [79, 80].

### Interleukin 6

Interleukin 6 (IL-6) is produced by a variety of cells, including DCs, macrophages, monocytes, T cells and vascular cells [81]. High levels of IL-6 correlate with increased BP and may be an independent risk factor for HT [81, 82]. In addition to this, the IL-6 level is reduced after treatment with Ang II-receptor blockade [83]. The increase of IL-6 is also observed in many models of experimental HT [84–86] strongly suggesting the essential role of IL-6 in HT. Treatment of mice with IL-6 not only increases vascular AT1 receptor expression but also induces vasoconstriction, oxidative stress and impairs endothelial function [87]. IL-6 mediates elevation of superoxide production and endothelial impairment by affecting NO-cGMP signalling pathway [88]. Furthermore, IL-6 has been reported to play an important role in VSMC migration and proliferation leading to vascular medial hypertrophy [89, 90]. Mice lacking IL-6 are protected against the action of Ang II and stress-induced HT [81, 85, 91]. Moreover, IL-6 promotes polarization of CD4+ T cells to produce IL-17 [92].

### Interleukin 17

Interleukin 17 (IL-17) is produced mainly by the unique subpopulation of CD4+ cells called TH17. Additionally, production of IL-17 was reported in γ/δ cells, subsets of CD8+ T cells, some B cells and NK cells [93]. Several reports indicate that IL-17 contributes to CVD [94–96]. Plasma levels of IL-17 are increased in humans and animals with HT [97–100]. Administration of recombinant IL-17 in mice causes a modest elevation of BP in the absence of other hypertensive stimuli [98]. Furthermore, genetic deletion or pharmacological blockade of IL-17 protects animals against Ang II and DOCA salt-induced HT, oxidative stress and endothelial dysfunction [97, 100]. Nguyen et al. have shown that IL-17 induces phosphorylation of Thr495 on the eNOS in Rho/Rho-kinase-dependent manner leading to decrease in NO production and impairment of Ach-induced relaxation in ex vivo studies [98]. Moreover, IL-17 activates ECs as demonstrated by elevated expression of adhesion molecules and other immune cells chemoattractants [101]. Mice lacking IL-17 are protected against perivascular inflammation [100]. Recently, Harrison’s group has shown that T cells, and especially TH17 cells, play crucial role in enhanced collagen deposition in adventitia and aortic stiffening in experimental HT [72]. It is evident that Il-17 induces expression of mRNA for collagens in p38MAP-kinase-dependent fashion leading to excessive collagen deposition and loss of aortic compliance [72]. Finally, IL-17 can induce or synergize effect of other pro-inflammatory cytokines [102] leading to perturbation between pro- and anti-inflammatory factors.

### Interleukin 10

In contrast to previously mentioned cytokines, interleukin 10 (IL-10) possesses anti-inflammatory properties. IL-10 not only suppresses production of TNF-α, IFN-γ and IL-6 by various immune cells [103], but also blocks the activity of pro-inflammatory transcription factors such as NF-κB [104]. It is produced by T cells, mainly Treg, DCs and macrophages [105, 106]. The IL-10 -627C/C polymorphism, associated with increased expression of IL-10, reduces an incidence of HT in Russian Tatars [107]. Hypertensive patients treated with AT1 receptor blockers or ACE inhibitors are characterized by an elevated serum IL-10 level [108]. Furthermore, there is much evidence that IL-10 blunts high BP in experimental models of HT, including preeclampsia [42, 109, 110]. Mice lacking IL-10 exhibit enhanced HT, endothelium dysfunction and increased superoxide production in response to Ang II compared with wild-type animals [63]. Similarly, incubation of the IL-10^−/−^ vessels with Ang II reduces their relaxation and enhances superoxide production as compared to wild-type vessels *ex vivo* [111]. On the other hand, administration of IL-10 or an antioxidant can restore Ang II-induced endothelial dysfunction [112]. IL-10, acting on ECs, upregulates expression, activity and phosphorylation of eNOS [113] and further inhibits activation of p38 MAP-kinase, which stimulates production of pro-inflammatory cytokines and regulates NADPH oxidase activity [114, 115].

## Clinical Evidence Linking Immune System and Hypertension

As stated above, experimental HT is associated with activated immune cells and their depletion/reduction very often results in normalization of BP. Based on the fact that immunosuppressive therapy is not currently clinically justified in patients with HT, we cannot countercheck these experimental observations directly. However, there is increasing evidence supporting the role of an immune component in the pathogenesis of HT in humans.

The third National Health and Nutrition Examination Survey (NHANES III) analysed data from 5626 participants and revealed a higher number of circulating leukocytes in hypertensive than in normotensive participants as well as the correlation between their number and systolic BP [116]. Increased BP is also observed in patients after infusion of allo-activated T cells during cancer treatment [117]. Conversely, HIV-infected patients have lower incidence of abnormally high BP [118]. Furthermore, immunosuppressive agents, which are not nephrotoxic, can reduce the prevalence of clinical HT [119, 120]. Reduction of BP with combination of telmisartan and rosuvastatin is related to a decrease of TH17/Treg ratio and of proinflammatory cytokines in hypertensive patients with carotid atherosclerosis [121]. Also, hereditary neutropenia, observed in Yemenites, causes small but statistically significant decline in systolic and diastolic BP in comparison to non-Yemenites population [64].

Increased BP in humans is associated with elevation of many pro-inflammatory mediators produced by immune cells such as TNF-α, IFN-γ, IL-6, IL-17 and decrease of anti-inflammatory cytokines like IL-10 [66, 69, 81, 82, 98]. This is also supported by the fact that polymorphisms of TNF-α and IL-6 are associated with human HT [122, 123].

Importantly, pro-inflammatory cytokines, mentioned above, serve not only as markers of inflammation but also confer risk for many CVD.

## Other Risk Factors Affecting Development of Hypertension and Their Link with Immune System

Physiological stress is a significant factor for many CVD [124]. Moreover, sympathetic nervous system participates in development of HT [11, 12]. Marvar et al. have demonstrated that repeated daily stress elevates BP in mice. This was associated with activation of the immune system i.e. increase of circulating T cells expressing CD69 and CD44^high^ markers. Moreover, Ang II-infused mice exposed to chronic stress displayed greater BP than non-stressed animals. In contrast, repeated stress did not affect BP in T and B cells depleted RAG1−/− mice [125].

Another risk factor for HT is obesity, which is also associated with chronic inflammation, vascular remodelling and endothelial dysfunction [126, 127]. Cells of both the innate and adoptive immune system reside in adipose tissue and participate in these pathophysiological changes [128]. Moreover, immune cells produce cytokines that can modulate expression of classical adipokines. Production of anti-inflammatory adiponectin can be inhibited by pro-inflammatory mediators such as TNF-α, IL-6 and IL-17. At the same time, pro-inflammatory cytokines can induce production of leptin which is critical in the development of obesity [33•].

Some studies have shown an association between air pollution exposure and the risk of HT [129]. Exposure to PM_2.5_ (particulate matter <2.5 μm) and ozone increases blood pressure in humans [130]. Similarly, PM_2.5_ exposure in mice induces endothelial dysfunction and mild elevation in BP, which is associated with increased levels of inflammatory cytokines in blood (TNF-α, IL-6). In addition to this, there is also an increased number of activated macrophages in adipose tissue and increased vascular adhesion of monocytes [131]. Finally, pollutant-induced oxidative stress may affect vascular function [132].

## Micro-RNA as Novel Immune Players in Hypertension

Micro-RNAs (miRNAs) are implicated in the intricate control of genes throughout the body; therefore, it is understandable that they can be associated with immune mechanisms of hypertension (Fig. 1). MiRNAs are 18–22 nucleotides in length and do not code for a gene but may affect expression of other target genes. This is achieved through a negative regulation via binding of its complex (the RNA-induced silencing complex) to the 3′ untranslated region (3′ UTR) of its target messenger RNA (mRNA). Sixty percent of protein coding genes have miRNA target sites in their 3′ UTR, and a single miRNA may impact the expression of multiple mRNAs. MiRNAs are involved in both the cellular and clinical manifestations of various CVD diseases such as HT [133], coronary artery disease [134] and cardiac hypertrophy with subsequent heart failure [135]. Further to this, miRNAs are implicated in the immune system in the context of chronic immune disorders [136–139]. Two miRNAs are discussed, which are considered to be pro-inflammatory in nature and demonstrated associations with chronic inflammatory arthritides as well as with cardiovascular manifestations of HT.

### miR-21

Micro-RNA 21 is an extensively investigated miR in the context of the cardiovascular system [140•]. MiR-21 is elevated in peripheral blood mononuclear cells (PBMCs) of hypertensive patients and is associated with the degree of LVH detected in this group [141]. In coronary artery disease, McDonald et al. demonstrated that eliminating miR-21 reduces in stent restenosis via anti-inflammatory effects of M2 macrophages, lowering number of CD3+ T cells and a reduction of Ly6c+cells level [134] (which differentiate to become the pro-inflammatory M1 phenotype). It is widely accepted that nitric oxide synthase is a marker for M1 macrophages and subsequently M1 cells produce ROS. This response was likely evolved to kill pathogens in the micro environment. That being said, it is now known that ROS elevate BP and activate pro-fibrotic signalling pathways as reviewed in many excellent reviews including this piece by Montezano and Touyz [142]. It is noteworthy that iNOS is not expressed by macrophages in humans; however, a clinical study has shown a relationship between elevated miR-21 and endothelial dysfunction with an associated reduction in NO and eNOS levels in hypertensive patients [143]. miR-21 can modulate T cell responses including exerting influence on cytokine production [144]. Not only miR-21 is induced by T cell activation and enhances the T cell immune response [145] but also miR-21 has been shown to induce proliferation of CD4+ T cells in murine models of systemic lupus erythematosus (SLE) and in patients with SLE [146, 147]. Relating to the production of IL-17 from CD4+ cells, previously mentioned in this work, this could link miR-21 to the increased cardiovascular risk observed in patients with lupus and other inflammatory arthritides. Further to this, miR-21 could serve as a biomarker for CVD in this patient group. miR-21 is a positive regulator of the expression of the transcription factor, FOXP3 [148]. As previously mentioned, Treg cells express FOXP3 and there is an association between a lower number of Treg cells and HT [38, 39]. This conflicts with the previously understood concepts that miR-21 is associated with pathological processes seen in elevated BP suggesting the full mechanism is not yet known, thus bringing together the development of HT, inflammatory cell lines, the coronary arterial disease sequelae of HT and miR-21.

### miR-155

The endothelium is the single-cell surface layer lining the entire vascular tree, and inflammation, the response to injury, is observed in the endothelium in HT. The endothelium is the source of eNOS, which underpins much of the vascular sequelae of HT. miR-155 reduces eNOS expression [149]. Mir-155 is regarded as a pro-inflammatory miR and is necessary for normal B cell and T cell function. In miR-155 KO mice, it has been shown that it is implicated in the regulation of many genes including chemokines and cytokines [150, 151]. Further to this, TNF-α increases miR-155 expression with subsequent negative regulation of eNOS [133, 152]. TNF-α has also been shown to increase miR-155 expression in macrophages and monocytes during an inflammatory response [153] helping to explain this phenomenon in HT. In short, the effects of TNF-α discussed earlier in this paper are closely mediated by miR-155. MiR-155 has been shown to regulate the AT1 receptor in rat cardiomyocytes [154], rat aortic adventitial-derived fibroblasts [155] and in human PBMCs where it was also found to correlate with BP [151]. Interestingly, rare allele of rs5186 polymorphism, located in the AT1 receptor, blocks adhesion of mir-155 to the AT1 receptor 3′ UTR [156]. This observation may explain that rs5186 was a risk factor for HT in several epidemiological studies, although overall, the clinical effect of this polymorphism is unclear and further research, taking into account different subpopulations of patients with HT, are needed to verify its role in HT [157]. MiR-155 level in aortic tissue of adult spontaneously hypertensive rats negatively correlates with the blood pressure [158]. This has translated into a human study that examined miR levels in PBMCs and demonstrated lower circulating pro-inflammatory cytokines including TNF-α in patients who had downregulation of miR-155 [159]. It has been shown that the 5p strand of miR-155 is upregulated in fibroblasts in the synovial fluid taken from patients with rheumatoid arthritis [160], suggesting that miR-155 plays an important role in a chronic inflammatory response. In summary, miR-155 is another miR, which provides an association between inflammatory arthritis and HT. Moreover, miR-155 is associated with hypertension and is implicated in a pro-inflammatory phenotype. It is likely that these effects may have an impact in either HT onset or severity.

## Conclusions

In summary, hypertension is associated with significant activation of immune and inflammatory systems and shares several functional differences with other immune-mediated diseases. Low-grade inflammation is prominent and further understanding of specific cytokine and chemokine milieu, similarities and differences between hypertension pathogenesis and atherosclerosis will shed a light on possible new therapeutic strategies to limit vascular and renal complications of HT. Indeed, in experimental models, immune-targeted therapies prevent vascular and renal damage and can alleviate hypertension. Evidence in humans is now urgently needed.
